# The need for translation and cultural adaptation of audiology questionnaires to enable the development of hearing healthcare policies in the Pacific Islands: a Samoan perspective

**DOI:** 10.1186/s13690-021-00606-3

**Published:** 2021-05-18

**Authors:** Annette Kaspar, Sione Pifeleti, Carlie Driscoll

**Affiliations:** 1 ENT Department, Tupua Tamasese Meaole Hospital, Ministry of Health, Apia, Samoa; 2 Audiology Division, School of Health and Rehabilitation Sciences, University of Queensland, Brisbane, Australia

**Keywords:** Translation, Cultural adaptation, Hearing, Questionnaire, Pacific Islands, Policy development

## Abstract

**Background:**

Translation and cultural adaptation of health resources is an integral part of good health-policy development and health program implementation. As part of our efforts to address ear disease and hearing loss in the Pacific Islands, we promote the translation an cultural adaptation of hearing-related questionnaires into local languages and cultural contexts. The Pacific Islands have among the highest rates of ear and hearing disorders in the world and, given the scarcity of ear/hearing health professionals in the region, a public health approach that uses appropriately translated ear/hearing health resources is highly recommended to tackle this health issue.

Although formal translation and culturally adaption of hearing-related questionnaires may seem a cumbersome process, the aim of this commentary is to illustrate the potential benefits of translating two audiology questionnaires for our use in Samoa. We have carefully selected questionnaires that will serve multiple purposes (i.e., clinical, epidemiology, monitoring and evaluation, evidence-based health policy formulation and implementation), thus making the process ultimately beneficial and worthwhile.

**Main body:**

The leading cause of preventable hearing loss among Samoan adolescents and young people is excessive noise exposure to recreational and environmental noise. The Youth Attitude to Noise Scale is a validated tool that assess knowledge and attitudes of adolescents towards recreational and environmental noise, and a Samoan version should provide preliminary data to guide health promotion activities for adolescents on noise-induced hearing loss.

The leading cause of hearing disability among older adult Samoans is age-related hearing loss. The Revised Hearing Handicap Inventory is a tool that assess the emotional and social/situational impact of hearing difficulty among older adults, and a Samoan version should provide preliminary data to guide the development of auditory rehabilitation services.

**Conclusion:**

Investment in quality translations and cultural adaptations of hearing-related questionnaires is essential for the development of audiology services that are relevant to their Pacific Island context. The use of formally translated audiology questionnaires in research studies will optimise data quality, leading to improved hearing health promotion activities, as well as provision of evidence for advocacy for public health noise policy legislation.

## Background

Translation and cultural adaptation of health resources is an integral part of good health-policy development and health program implementation. As part of our efforts to address ear disease and hearing loss in the Pacific Islands, we promote the translation an cultural adaptation of hearing-related questionnaires into local languages and cultural contexts [1]. The Pacific Islands have among the highest rates of ear and hearing disorders in the world [2] and, given the scarcity of ear/hearing health professionals in the region [3], a public health approach that uses appropriately translated ear/hearing health resources is highly recommended to tackle this health issue [4].

The Ear, Nose and Throat (ENT)/Audiology Department of the Tupua Tamasese Meaole Hospital in Samoa is currently engaged in a research project that aims to address ear/hearing disorders in the country through clinical, public health, and health promotion services. A review of the literature indicates that there are a number of validated audiology questionnaires that should prove useful to achieve our goals. The advantages of questionnaires in a low-resourced context such as Samoa include: administration does not required ENT/Audiology specialists, rapid assessment possible, procurement of data to inform the development of public health initiatives, procurement of data to inform the development of ENT/Audiology services, and a tool that enables procurement of baseline data, followed by updated data at a later time for monitoring and evaluation purposes. To optimise these objectives, it is imperative to translate and culturally adapt selected questionnaires for our Samoan context.

Although formal translation and culturally adaption of hearing-related questionnaires may seem a cumbersome process, the aim of this commentary is to illustrate the potential benefits of translating two audiology questionnaires for our use in Samoa. We have carefully selected questionnaires that will serve multiple purposes (i.e., clinical, epidemiology, monitoring and evaluation, evidence-based health policy formulation and implementation), thus making the process ultimately beneficial and worthwhile.

## Main text

### Addressing noise-induced hearing loss from recreational and environmental noise exposure: youth attitude to noise scale

The leading cause of preventable hearing loss among Samoan adolescents and young people is excessive noise exposure to recreational and environmental noise. According to the World Health Organization, nearly 50% of people aged 12–35 years worldwide are at risk of developing permanent noise-induced hearing loss [5]. In addition to causing permanent hearing damage, excessive noise exposure may also lead to adverse consequences such as tinnitus, headaches, and poor sleep and concentration [5]. The Samoan ENT/Audiology Clinic therefore selected “Enjoy your music at safe listening volumes” as one of our messages for World Hearing Day 2020.

In order to build on this hearing health message, we are collaborating with the health promotion department to develop health education activities specifically for adolescents about recreational noise exposure. The Youth Attitude to Noise Scale (YANS) is a validated tool that should assist our team in this endeavour, given that it assess knowledge and attitudes of adolescents towards recreational and environmental noise [6]. It consists of 19 statements requiring a response from the respondent on the 5-point Likert scale (Strongly Disagree/Disagree/Neutral/Agree/Strongly Agree). There are seven statements regarding noise-related attitudes associated with the culture of youth (i.e., music festivals), five statements investigating attitudes to daily noise (i.e., traffic noise), four questions concerning the skill to concentrate in noisy environments (i.e., school), and three questions assessing willingness to advocate for changes in noisy environments (i.e., school).

A review of the literature found formal studies on the translation and cultural adaptation of the YANS to Brazilian Portuguese [7] and Chinese [8]. We have begun preparations to perform similar work in collaboration with the Centre for Samoan Studies at the National University of Samoa, and will follow the recommendations outlined in the paper by Hall et al. [1]. A Samoan version of the YANS will be administered to adolescents during the ENT/Audiology Clinic outreach visits to secondary schools in Samoa. All data handling and analysis will be performed by the research audiologist (author AK) in conjunction with her Australian colleagues at the University of Queensland (Brisbane, Australia). A manuscript will be prepared for publication by the research audiologist (author AK) in collaboration with the Samoan ENT/Audiology Clinic team.

The results of the Samoan YANS research study should provide preliminary evidence for the development of health promotion activities for adolescents on noise-induced hearing loss. It should also prove a valuable resource for hearing health advocates in their efforts to influence implementation of public health noise policies. For example, the results of the study may lead to initiatives that reduce noise levels in the educational setting, and thereby promote a more positive and improved learning experience. Following implementation of any noise-related interventions, the Samoan YANS should be repeated in order to assess the impact of initiatives on the listening experience of students: in this way, the translated questionnaire serves an on-going purpose of monitoring and evaluation of health promotion programs for addressing permanent hearing loss among adolescents in Samoa from excessive recreational/environmental noise exposure.

### Assessing the impact of hearing disability on quality of life among older adults: revised hearing handicap inventory

The leading cause of hearing disability among older adult Samoans is age-related hearing loss. According to the World Health Organization, approximately a third of adults over 65 years of age experience disabling hearing loss [2]. There are no epidemiology studies from the Pacific Islands on the prevalence and degree of hearing loss among older adults in the Pacific Islands, and data are needed to guide the development of appropriate ENT/Audiology services for older adults. The World Health Organization has published a survey protocol to enable the collection of audiometric data for such purposes, however, such a survey may be difficult to perform in our resource-limited setting. A questionnaire that evaluates the self-reported functional hearing disability associated with age-related hearing loss is an alternative option that should prove useful in our context [9]. Indeed, it may be more appropriate and informative to assess the impact of hearing loss on quality of life rather than attempt formal epidemiological studies of hearing level measurements.

The Revised Hearing Handicap Inventory (RHHI) emerges as a suitable candidate for our situation in Samoa [10]. It consists of 18 questions requiring a “Yes”, “Sometimes”, or “No” response from the respondent. The questions investigate the social/situational impact of hearing difficulty, as well as the emotional impact of hearing challenges.

Similar to the discussion above, we aim to develop a Samoan version of the RHHI in collaboration with the Centre for Samoan Studies at the National University of Samoa in accordance with the procedures described by Hall and colleagues [1]. The Samoan version of the RHHI will then be administered to older adults during the ENT/Audiology Clinic outreach visits to urban, rural and remote villages in Samoa. This will be done with the support of the Samoan Office of the World Health Organization. Again, all data handling and analysis will be performed by the research audiologist (author AK) in conjunction with her colleagues at the University of Queensland (Brisbane, Australia), and a manuscript will be prepared for publication in collaboration with the Samoan ENT/Audiology Clinic team.

The results of the Samoan RHHI research study should provide preliminary evidence on the social and emotional impact of hearing disability among older adults in Samoa. It should also inform the development of ENT/Audiology services for this population. Auditory rehabilitation services are limited but evolving in Samoa, and the results of the RHHI study should guide our clinic towards the most effective strategy for improving the listening enjoyment of older adults. Samoan culture is characterised by collectivism, and it may be more acceptable to the community to develop hearing health policies that promote expenditure on technology that benefits the entire community (i.e., loudspeakers for village meetings) rather than individual hearing health plans (i.e., hearing aids). Similar to the discussion above, the Samoan RHHI study should be repeated at a later time to assess the effectiveness of any interventions: again, the translated questionnaire will serve an on-going purpose of monitoring and evaluating the hearing health of older adults in Samoa.

## Conclusion

Investment in quality translations and cultural adaptations of hearing-related questionnaires is essential for the development of audiology services that are relevant to their Pacific Island context. The use of formally translated audiology questionnaires in research studies will optimise data quality, leading to improved hearing health promotion activities, as well as provision of evidence for advocacy for public health noise policy legislation.

## Data Availability

Not applicable.

## Abbreviations

ENT: Ear, Nose & Throat
RHHI: Revised Hearing Handicap Inventory
YANS: Youth Attitude to Noise Scale
